# Is balanced salt solution really superior to ringer lactate for phacoemulsification?

**DOI:** 10.4103/0301-4738.60073

**Published:** 2010

**Authors:** Parikshit Gogate, Madan Deshpande

**Affiliations:** H.V. Desai Eye Hospital, Pune, India

Dear Editor,

The article by Vasavada *et al*. on the comparison of the use of Ringer's lactate (RL) and a balanced salt solution (BSS) on the postoperative outcomes of phacoemulsification, by a randomized control trial, has added *in vivo* evidence about the superiority of BSS plus.[1]

But except for the first postoperative day, there was no significant difference between the use of two fluids for phacoemulsification. It would have been better if the authors had also showed the visual acuity results on the first postoperative day. We presume that there were no intraoperative complications. A difference of 25 μm of corneal swelling might have been “statistically” significant, but was it “clinically” significant in terms of suboptimal visual acuity? A one-week follow-up would also have helped. An Indian-made RL costs about Rs. 25 only (< $0.5) compared to Rs. 2800 ($62) for BSS plus. If we were to calculate a cost-benefit ratio, would a single-line visual acuity difference on the first postoperative day justify such an increased cost for most patients? Even Indian-made BSS are more than four times as costly as the RL. Also, the aqueous turnover time in the anterior chamber is less than 24 h. Hence the irrigating solution would not make any difference beyond the first few days.

BSS plus definitely would have incrementally helped in complicated cataracts, very hard cataracts, patients with poor endothelial cell counts, very old patients, and also where such high-viscosity devices were not available. The authors need to be congratulated for such less endothelial cell loss,[1] compared to other studies.[2,3] This might be due to the operating surgeon's vast experience, selection of cataracts, and use of high-viscosity agents such as Provisc™ and Viscoat™.

In a country like ours, operating surgeons need to choose their consumables rationally, not just the best available, but rather optimally available.[4] It can be tailored for each cataract surgery. Ruit *et al*. had found no significant difference in corneal thickness and visual acuity, even on the first postoperative day, when comparing manual small incision cataract surgery and phacoemulcification.[3] A BSS is better, but it can only be called “statistically,” not “clinically” superior to the RL, unless we have evidence to the contrary.
